# The New Zoonotic Malaria: *Plasmodium cynomolgi*

**DOI:** 10.3390/tropicalmed6020046

**Published:** 2021-04-05

**Authors:** Alexander Bykersma

**Affiliations:** MBBS, Cairns Base Hospital, 165 Esplanade, Cairns, QLD 4870, Australia; alex_bykers@hotmail.com

**Keywords:** Plasmodium cynomolgi, malaria: simian malaria, zoonotic infections

## Abstract

*Plasmodium cynomolgi* is a simian malaria parasite that has been a central model parasite since it was first described in 1907. Recently it has made the zoonotic jump and started naturally infecting humans. In this paper, the interactions between *Plasmodium cynomolgi* and humans, the environment and the non-human animal intermediates or definitive host will be discussed, with a particular focus on the clinical implications of infection and approaches to management of this novel zoonotic parasite.

## 1. Introduction

Malaria is a haemoparasitic disease caused by the apicomplexan protozoa of the plasmodium species that is transmitted via the infected female *Anopheles* mosquito. Malaria poses a significant public health burden across the world, impacting an estimated 219 million people across the world, being the most endemic in the African Region and South East Asia [1,2,3,4]. Traditionally, there are 4 plasmodium species that cause natural human malaria infection, those being *Plasmodium falciparum, Plasmodium vivax, Plasmodium ovale, and Plasmodium malariae.* However, numerous simian plasmodium species have now been reported to naturally infect humans [5,6]. 

The list of experimentally transmitted simian malaria species that have been known to cause infection in humans via a mosquito vector includes but is not limited to *Plasmodium cynomolgi, Plasmodium simium, Plasmodium inui, Plasmodium brasilianum* and *Plasmodium knowlesi* [7,8,9,10,11,12,13]. Such interest in potentially zoonotic malaria began following 1960, where two laboratory workers became accidentally infected with *Plasmodium cynomolgi* via an *Anopheles* mosquito [14]. 

*Plasmodium cynomolgi* predominately causes malaria in macaque monkeys but is also known to cause experimental and rare natural zoonotic infections in humans [15]. There are considerable morphological and biological comparisons between *P. cynomolgi* and its sister taxon *P. vivax*. Both of which have exoerythrocytic dormant stages in the liver with associated hypnozoites; they favour infecting reticulocytes and have the Schuffner’s dot modified infected erythrocyte membrane [16]. Although *P. vivax* has a much larger public health burden than *P. cynomolgi*, *P. vivax* cannot be cultured in vitro, whereas *P. cynomolgi* can be [15]. Nevertheless, due to their similarities, *P. cynomolgi* represents an ideal surrogate for studying the features of *P. vivax*. This has already been done, as *P. cynomolgi* was imperative in identifying primaquine as a management option, and it will continue to provide opportunities to develop new treatment options against dormant liver hypnozoites [17]. 

The inability to robustly culture *P. vivax* has led to the majority of the information about its lifecycle and biology being derived from the culture and study of *P. cynomolgi* [18,19]. There are currently no reports of natural human to human transmission of *P. cynomolgi*, and thus, the macaque monkey should still be considered as the natural intermediate host. By definition, making *P. cynomolgi* a zoonotic infection. 

The currently researched risk factors for infection with zoonotic malaria include the male sex, contact with macaque monkeys, agricultural expansion, and forest fragmentation. Geographical elevation and use of insecticide were found to be protective factors against zoonotic malaria infection [20]. Other generalised malaria risk factors that one can assume also impact zoonotic malaria are poor environmental management to decrease *Anopheles* larval stores, surrounding stagnant water, not sleeping under insecticide treated mosquito nets, and climate of the area [21,22]. 

## 2. Clinical Features and Past Human Infections

Despite the experimental human infections with *P. cynomolgi*, the first naturally acquired human *P. cynomolgi* infection occurred at the start of 2011 in a 39-year-old Malay woman from the east coast of Peninsular Malaysia [5]. Since then, there have been another symptomatic infection of a tourist travelling to Malaysia, cases in Malaysian Borneo, 2 additional Malaysian cases and multiple asymptomatic infections in Cambodia [6,23,24,25,26]. Prior to this, the clinical symptoms and disease profile were only known from experimental infections, which showed that *P. cynomolgi* was characterised by myalgia, cephalgia, anorexia, nausea and fever [27,28]. These cases had an associated 7–16 day prepatent period and an incubation period of approximately 7–16 days [27,28]. In the reported symptomatic natural infections, the patients experienced cyclical fevers, myalgia, general malaise, cephalgia, and abdominal pain that were described as generalised non-specific flu-like syndrome [5,6]. 

During the diagnosis of these symptomatic patients, collected blood samples were analysed via microscopy and molecular diagnostic methods. The blood films had some morphological features of *P. vivax*, which included amoeboid trophozoites, mature microgametocytes that are round and resemble those of *P. vivax* at a similar developmental stage, and growing trophozoites with clearly visible Schuffner’s dots [16]. However, when molecular species detection testing was performed using a polymerase chain reaction (PCR), the four commonly known human malaria species were negative for *P. falciparum, P. vivax, P. ovale* and *P. malariae,* as was testing for *P. knowlesi.* Further testing was positive for *P. cynomolgi* [5,6]. This highlights the importance of PCR in the diagnosis of *P. cynomolgi* seeing that it is morphologically indistinguishable with *P. vivax* when assessed under a microscope. As *P. cynomolgi* co-infection with other malaria species has been reported in the literature, this creates a potential for *P. cynomolgi* underdiagnosis [26]. A reason for this being that *P. cynomolgi* PCR is not routinely conducted; thus, all co-infections would be attributed solely to the other malaria species that is a part of the co-infection. However, it would also not be pragmatic to run a *P. cynomolgi* PCR for all patients across the globe with an infection from one of the more common species of malaria, due to the exceeding rarity of *P. cynomolgi*.

The other studies of human patients with *P. cynomolgi* infections showed that infected individuals are nearly all asymptomatic, with only few experiencing mild symptoms [8,23,24,25,26,27]. Due to the limited disease severity of *P. cynomolgi* and its morphological similarity to *P. vivax* which makes accurate microscopic speciation difficult, it is likely that the true incidence rates of *P. cynomolgi* is likely to be significantly higher than it is currently believed to be. In fact, many of the patients in these studies with *P. cynomolgi* mono-infection would have been incorrectly diagnosed as *P. vivax* infections if only microscopy or rapid diagnostic tests were used, without ever employing the use of molecular methods [5,6,25,26]. 

## 3. Medication Management

The aims of malaria management are to decrease the immediate risk to the host, lower the peripheral asexual parasitaemia, avoid relapses by eradicating dormant stages, and to ultimately prevent disease transmission to other individuals in the community [29]. Both *P. vivax* and *P. cynomolgi* produce dormant liver stage hypnozoites that add another layer to the treatment of patients infected with these parasites, because they could potentially be at risk of an infection relapse following the first initial infection. Currently, there is no single medication that exists which can eradicate *P. vivax* or *P. cynomolgi* at each of their respective lifecycle stages. Therefore, it is recommended to use combination therapy. Combination therapy involves utilising primaquine in those that do not have a glucose-6-phosphate dehydrogenase deficiency in addition to the chosen primary anti-malarial. It should be noted that there have not been any clinical trials involving the treatment of *P. cynomolgi* in naturally infected humans, due to the rarity of the condition. As a result, the most logical approach to treating future *P. cynomolgi* infections in humans should be based off the treatment options for its sister taxon *P. vivax.*

## 4. Ecological Management

The relationship between the *P. cynomolgi* parasite and its physical surroundings provides another potential opportunity for future intervention and disease control through integrated vector management. The overarching concept of integrated vector management is that vector control should not occur through only one mode of intervention, but rather should have a multi-layered approach. 

One of the foremost methods for vector control is to utilise insecticide products [30]. Commonly this is performed via indoor spraying and/or the use of mosquito nets that have been treated with an insecticide, that can decrease malaria transmission by up to two orders of magnitude [31,32]. Unfortunately, when utilised as the single intervention for a geographical area with a high entomological rate, the insecticide programs are not sufficient enough [33,34]. This is why a combination approach should be used, involving both the indoor residual spraying and the use of insecticide treated nets in the home. Such insecticide treatments can have a cumulative effect on vector transmission inhibition, by impeding on the vectors lifecycle which thereby reduces the period that the mosquito is able to infect other individuals [35]. The utilisation of insecticide treated nets and indoor residual spraying in unison has led to the majority of malaria burden reduction achieved recently in low and middle-income countries [36]. 

The use of insecticide chemicals is often considered to be a pillar in an ecological vector control strategy; however, the tendency for vectors to evolve diverse resistance mechanisms is well documented [37]. Malaria vector insecticide resistance has been witnessed to all of the common classes of insecticide [38]. A proposed way to combat the issue of insecticide resistance in *Anopheles* mosquitoes is to supersede the current treated nets with the newer models that have a mixture of insecticides as well as have alterative insecticides utilised in them [39]. 

The World Health Organisation framework for ecological control involves environmental modification, environmental manipulation, and strategies that reduce contacts between vectors and humans [35,40]. To implement environmental modification measures, a large capital investment is usually required, but it does develop lasting infrastructure that includes improved water, irrigation and dam technologies. This is because these programs augment the environmental movement and levels of water that can change the vector’s natural habitats and have been shown in various parts of Asia to disrupt the *Anopheles* breeding cycle [35,41,42]. In contrast, environmental manipulation allows for faster methods to impact the vector habitat. This can be achieved through the preventing shady areas for mosquitos that prefer a shady environment by removing overgrowing vegetation and aquatic weeds and by altering the drainage patterns of an area to avoid stagnant water. Similarly, if a vector prefers a sunny environment, then, more covered over areas can be created where they would normally live. Strategies that reduce contacts between vectors and humans should also be incorporated into the multimodal approach to malaria vector prevention. This could occur by simply ensuring that humans settle in an area far away from vector breeding grounds. Livestock and animal pens should be separated from human dwellings to prevent the vectors attracted to the animals opportunistically feeding on humans. Finally, people should utilise mosquito nets and window screens around the home. 

An interesting novel approach to environmental modification for vector control would be using fish that eat the larvae of mosquitos, in order to control the vector population. This method is more targeted towards the mosquito larvae and thus minimises any environmental damage from insecticides. The most common example of larvivorous fish that can be used is *Bacillus thuringiensis israelensis* and *Bacillus sphaericus* [43]. 

## 5. Conclusions

*Plasmodium cynomolgi* is the new and exciting zoonotic malaria to naturally infect humans. Simian malaria species may become more common in the future and even more worryingly, the accurate diagnosis may potentially be missed due to the morphological and biological similarities between *P. cynomolgi* and *P. vivax*. The significance of *P. cynomolgi* that is transmissible cannot be ignored given the increasing identification of infection. An integrated treatment program to mitigate the emerging public health threat must be multi-layered and incorporate both pharmacological and ecological approaches.
